# Distribution pattern and change prediction of *Phellodendron* habitat in China under climate change

**DOI:** 10.1002/ece3.10374

**Published:** 2023-08-25

**Authors:** Yanghui Zhao, Yafeng Wen, Wenqian Zhang, Chuncheng Wang, Yadan Yan, Siwen Hao, Donglin Zhang

**Affiliations:** ^1^ College of Landscape Architecture Central South University of Forestry and Technology Changsha China; ^2^ Hunan Big Data Engineering Technology Research Center of Natural Protected Landscape Resources Changsha China; ^3^ Yuelushan Laboratory Carbon Sinks Forests Variety Innovation Center Changsha China; ^4^ Department of Horticulture University of Georgia Georgia Athens USA

**Keywords:** climate change, geographical distribution, MaxEnt model, parameter optimization, *Phellodendron*

## Abstract

*Phellodendron* has always been of great significance in promoting human health and ecological restoration. However, human activities and climate change have severely affected habitat, population dynamics and sustainable use of *Phellodendron*. Little is known about the geographical distribution pattern and their responses to climate change of *Phellodendron*. In order to reveal the impact of climate change on *Phellodendron*, we conducted a study based on natural distribution data of two species (297 occurrence points), 20 environmental factors, and an optimized MaxEnt model. Our results identified the main environmental factors influencing *Phellodendron*, predicted their potential geographical distribution, and assessed migration trends under climate change in China. Our analysis showed that *Ph. amurense* and *Ph. chinense* have potential suitable habitats of 62.89 × 10^4^ and 70.71 × 10^4^ km^2^, respectively. Temperature and precipitation were found to play an essential role in shaping the present geographical distribution of *Phellodendron* populations. Based on two future climate scenarios, we forecasted that the potential suitable habitat of *Ph. amurense* would decrease by 12.52% (SSP245) and increase by 25.28% (SSP585), while *Ph. chinense* would decline by 19.61% (SSP245) and 15.78% (SSP585) in the late‐21st century. The potential suitable habitats of *Ph. amurense* and *Ph. chinense* would shift to northward and westward, respectively. Hydrothermal change was found to be the primary driver of the suitable habitat of *Phellodendron* populations in the future. We recommend establishing nature reserves for existing *Phellodendron* populations, especially *Ph. chinense*. Our study provided a practical framework for the impact of climate change on the suitable habitat of *Phellodendron* species and guided regional cultivation, long‐term conservation, and sustainable use.

## Graphical abstract

Creating a scientific and practical technical framework for investigating the impact of climate change on the suitable habitat of Phellodendron vegetation. Temperature and precipitation are the key factors influencing the habitat changes of the two Phellodendron species. Revealing the potential habitat redistribution pattern of Phellodendron vegetation in response to future climate change and identifying hotspots of habitat degradation/expansion. Guiding regional cultivation, long‐term conservation, and sustainable use.

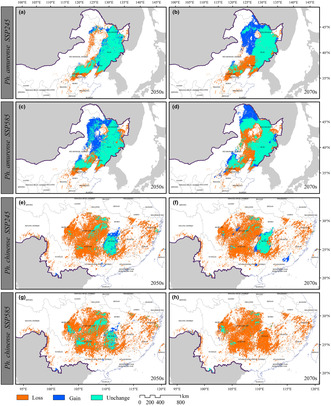

## 1 INTRODUCTION

Over the past several decades, climate change has become the primary driver of biodiversity loss and the most significant global ecosystem threat, overtaking habitat destruction (Leadley et al., 2010; Pereira et al., 2010). Understanding the impact of climate change on biodiversity and developing effective conservation strategies has become a pressing global issue for the international community (Bongaarts, 2019; Hooper et al., 2012; Ye et al., 2021). Rare and endangered plants are especially vulnerable to climate change and human activities, and they play a crucial role in alternative biodiversity indicators, highlighting the need for their conservation (Li, Chang, et al., 2019; Yang et al., 2021). Unfortunately, many rare and endangered plants face an increased risk of habitat destruction and extinction due to climate change and human activities (Pandey et al., 2019). Although many studies have explored the effects of climate change on rare and endangered plants through laboratory, field warming, and species diversity at different geographical scales (Li et al., 2021; Zhang, Willis, et al., 2017), which greatly improves the conservation efficiency and sustainable use of target plants, there are also some limitations. For example, field warming experiments can only concentrate on small areas and minimal environmental factors (Li, Chang, et al., 2019; Wang, Huang, et al., 2021), and phenology characteristics and distribution patterns vary greatly among species, with different influencing factors (Xie et al., 2021). Consequently, many rare and endangered plants still lack a comprehensive understanding of their response to climate change. Therefore, more studies are increasingly employing species distribution models (SDMs) to study the impact of climate change on the geographical distribution patterns of rare and endangered plants, utilizing more environmental factors (Bellard et al., 2012, 2014).

Species distribution models have been important for studying ecological issues between species and the environment under global environmental change (Bellard et al., 2012; Gaston, 2000). They have been widely used in biogeography (Ye et al., 2021) and conservation biology studies (Pandey et al., 2019), such as habitat conservation for endangered species (Li, Chang, et al., 2019). Various SDMs are utilized for predicting species distribution, including climate change experiment (CLIMEX), genetic algorithm for rule‐set production (GARP), ecological niche factor analysis (ENFA), the BIOCLIM model, and maximum entropy (MaxEnt) model (Guisan & Thuiller, 2005; Phillips et al., 2006; Zhao et al., 2021). Multi‐model inter‐comparison studies have shown that the MaxEnt model outperforms other SDMs in terms of high tolerance and high prediction accuracy (Muscarella et al., 2014; Radosavljevic & Anderson, 2014) and it is robust to real absence data, which can influence model outcome settings (Elith et al., 2010; Phillips et al., 2006, 2017). MaxEnt tests the patterns of occurrence (presence points) against randomly selected pseudo‐absence points (Paź‐Dyderska et al., 2021). Moreover, the MaxEnt model can simulate the complex nonlinear relationship between response and prediction variables for modeling (Warren & Seifert, 2011). As a result, many researchers prefer using the MaxEnt model for studying species distribution.

However, the drawback is that the MaxEnt model is often used with default or previously published parameters without considering the details of the algorithm or input parameters and all environment variables are incorporated in the model without distinction (Liu et al., 2019; Muscarella et al., 2014). These severely increase the model's complexity and execution time, causing the model's results to overfitting and deviation (Chen et al., 2019; Phillips & Dudík, 2008). To mitigate auto‐correlation among environmental variables, minimize model execution time, and alleviate data overfitting, previous studies have employed methods such as correlation analysis to screen environmental variables, ultimately selecting the optimal model based on the minimum AICc value. For instance, Sheppard (2013) demonstrated in a study of three new weeds that the deliberate and thoughtful selection of predictor variables can decrease model execution time and uncertainty in predicting species distributions. Li, Li, et al. (2018) utilized the MaxEnt model and employed the AICc criterion to determine the optimal parameter settings for feature combination (FC) as LQP and the regularization multiplier (RM) as 1.5. With these settings, they successfully predicted the potential distribution of *Quercus aliena* in China. Previous studies have confirmed how diverse environmental factors and parameters used in SDMs affect the accuracy of model prediction (Elith et al., 2010; Li et al., 2020; Zhao et al., 2021). Hence, selecting the optimal environmental factors and model parameters is crucial while using the MaxEnt model to study species distribution.

The *Phellodendron* belongs to the Tertiary relict plants of the Rutaceae family. It comprises deciduous trees and encompasses only four species, mainly distributed across Europe and Asia (Li, 2013). They are of utmost importance for regional ecological construction and are a source of drug material (Sun et al., 2019; Yang et al., 2016). With the discovery of medicinal value and the increasing market demand for *Phellodendron*, it has been over‐harvested, resulting in a sharp population decline. For instance, the wild *Ph. amurense* forest stock volume in Jilin and Heilongjiang provinces of China declined by 33.9 × 10^4^ m^3^ and 25.1 × 10^4^ m^3^, respectively, in the 5 years (Qin et al., 2006). More seriously, climate warming has significantly impacted the reproduction and survival of *Phellodendron* plants (Wang, Huang, et al., 2021; Yang et al., 2019). For example, the phenology characteristics of *Ph. chinense* have changed due to global warming, leading to a temporal and spatial mismatch between its flowering period and pollinators (Fan et al., 2021; Huang et al., 2012). This mismatch disrupts their synergistic relationship and reduces the number of individuals produced by sexual reproduction. The conservation of these species is deemed exceedingly imperative. Currently, wild populations of *Ph. amurense* and *Ph. chinense* are primarily found in China, with the distribution center also located there. However, China is and will continue to be a climate change hotspot in the future (Huang et al., 2020; Lu et al., 2020). Driven by climate change, plants may change their phenological or physiological responses or migrate to more suitable habitats to avoid being affected by adverse climatic conditions (Liao et al., 2020). To reveal the impact of climate change on the main distribution areas of *Ph. amurense* and *Ph. Chinense*, the study area was set in the northern and southern regions of China. We hypothesized that (1) the suitable planting area of *Ph. amurense* and *Ph. Chinense* will decrease, and (2) with a northward extension and migration due to warmer and wetter conditions in the future. Our study has four primary objectives:

1. Assessing the impact of different parameter settings on the performance of the MaxEnt model and investigating the influential environmental factors that affect the distribution of *Ph. amurense* and *Ph. Chinense*.
2. Projecting the potential suitable habitats and centroid migration of the two *Phellodendron* species under future climate change scenarios.
3. Uncovering the patterns of habitat redistribution in *Phellodendron* populations in response to climate change, identifying areas of habitat degradation/expansion.
4. Establishing a scientific and practical framework to assess the impact of climate change on the suitable distribution areas of the two *Phellodendron* species.

Our goal is to provide reference value and scientific guidance for their cultivation, conservation, restoration, and sustainable use.

## 2 MATERIALS AND METHODS

### 2.1 The species studied

**FIGURE 1:**
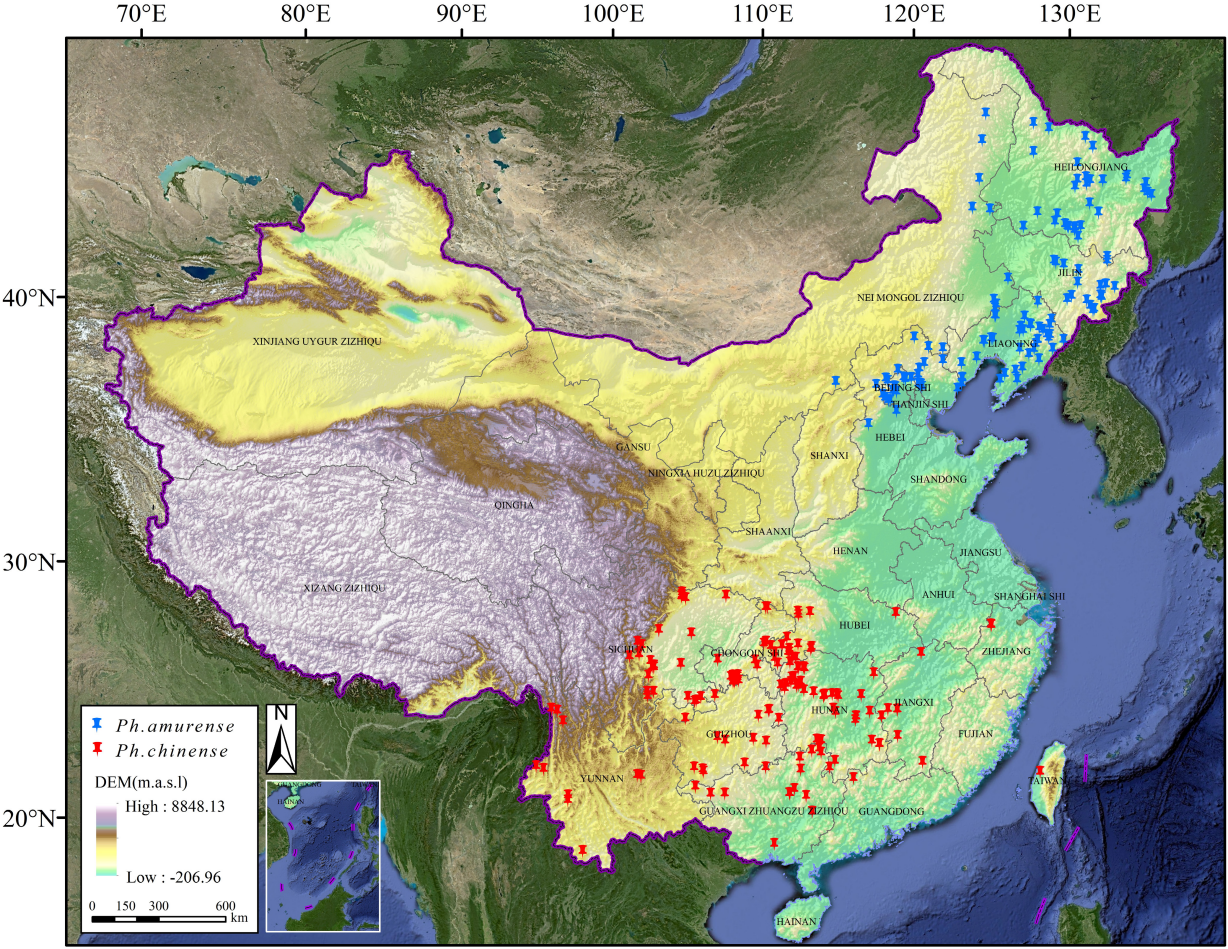
The spatial distribution of occurrence records of *Phellodendron amurense* and *Ph. chinense*. Base map were downloaded from the Standard map service of the Ministry of Natural Resources (http://bzdt.ch.mnr.gov.cn/; GS(2021)5454).

*Phellodendron amurense* and *Ph. chinense* are deciduous trees that have a monoecious reproductive system. The former is primarily distributed in East Asia, Central Asia, Eastern Europe and northern China, while the latter is distributed only in southern China. Among them, *Ph. amurense* is a light‐demanding plant that is tolerant of harsh winter conditions, characterized by a well‐developed root system and strong natural regenerative ability. It mainly grows in temperate coniferous forests and broad‐leaved mixed forests in northern China. *Ph. chinense* exhibits a preference for moist environments and is sensitive to high temperatures and water stress. Therefore, it mainly grows in the subtropical broad‐leaved forest region. The primary natural distribution ranges of *Ph. amurense* and *Ph. chinense* are located around 115°–136° E, 34°–46° N, and 95°–120° E, 17°–28° N, respectively (Figure 1).

### 2.2 Study area

The northern area lies east of the Greater Khingan Range and Qinghai‐Tibet Plateau, south of the Inner Mongolia Plateau, and north of the Qinling‐Huaihe River (Figure 1). It is primarily characterized by plains and plateaus, with a temperate monsoon climate, significant temperature fluctuations between winter and summer, and extreme minimum and maximum temperatures of approximately −40 and 30°C, respectively (Huang et al., 2018; Qin et al., 2006). The annual precipitation in this region ranges between 400 and 800 mm. The southern region of China is located south of the Qinling‐Huaihe River and east of the Qinghai‐Tibet Plateau (Figure 1). On the other hand, the southern area is situated south of the Qinling‐Huaihe River and east of the Qinghai‐Tibet Plateau, with a varied terrain that includes plateaus, basins, plains, low mountains, and hills. The hottest monthly average temperature is higher than 22°C, while the coldest monthly temperature is between 0 and 15°C, and the annual precipitation is approximately 1000–1500 mm (Yang et al., 2019; Zhang et al., 2016).

### 2.3 Methodological framework

**FIGURE 2:**
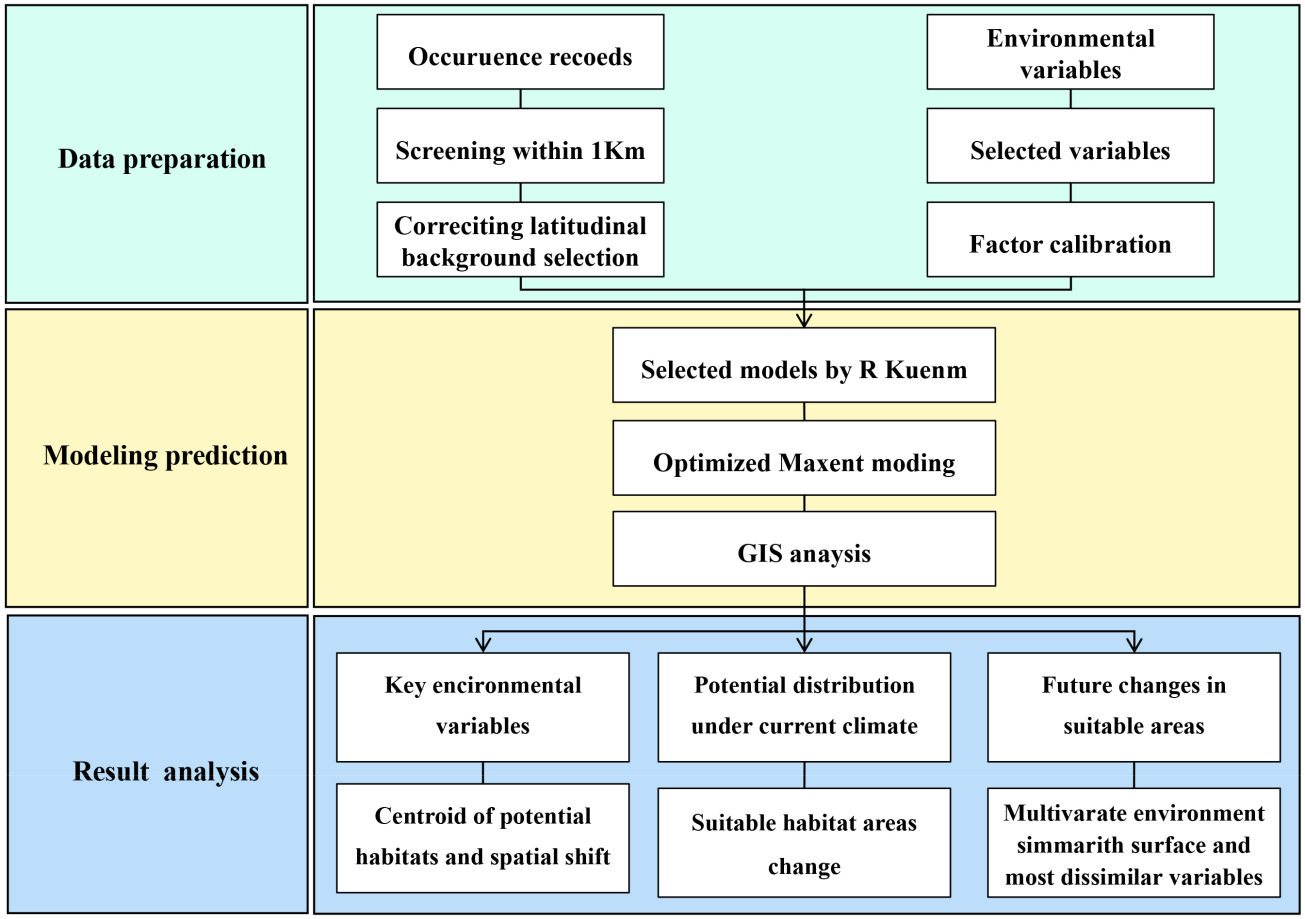
Flowchart of the methodological workflow for this study.

A visual representation in the form of a flowchart was constructed to encapsulate the complete methodology employed. This methodology centered around assessing the improvement in MaxEnt model performance before and after optimization and determining how changes in environmental variables could affect the geographic distribution of species and their suitable habitat in the wake of climate change (Figure 2).

### 2.4 Species distribution records

We adopted three methods to obtain complete information on the geographic distribution of *Ph. amurense* and *Ph. chinense* in China (after 2000). (1) Species occurrence databases. The distribution data of *Ph. amurense* and *Ph. chinense* were obtained by searching GBIF (Global Biodiversity Information Facility), NSII (The China National Specimen Information Infrastructure), and CVH (The Chinese Virtual Herbarium). (2) Scientific articles. We searched the Web of Science (WOS; https://www.webofscience.com/) and the China National Knowledge Infrastructure (CNKI; https://www.cnki.net/) database with Latin names of the species and only retained literature with precise field distribution site records. (3) Field investigation. From 2019 to 2021, we conducted field surveys of natural populations of *Ph. chinense* in Guizhou, China, and recorded each sample site's latitude, longitude, and habitat characteristics. Accordingly, we have gathered 938 comprehensive occurrence records of the targeted flora, encompassing 384 instances of *Ph. amurense* and 554 instances of *Ph. chinense*. To improve the reliability and accuracy of the species distribution data, we processed the distribution data of the target species as follows: Firstly, we imported species distribution data (384 instances of *Ph. amurense* and 554 instances of *Ph. chinense*), Google Maps (http://www.gditu.net/), and 2020 land use data (https://www.resdc.cn/) into ArcGIS software for overlay analysis to eliminate planting areas (residential areas), redundant, and inaccurate data (on water bodies, buildings, and roads). Secondly, we have employed the R software package “spThin” to eliminate the duplicate data to avert spatial auto‐correlation and reduce superfluous data. Thirdly, we have utilized ENMTools, a niche model analysis software that has the ability to associate species distribution data with environmental data for comparative analysis to retain only one accurate distribution data point within the same environmental data raster. It can effectively avoid excessive concentration of species distribution data that may lead to model overfitting. We have kept only one distribution point for each 2.5′ × 2.5′ grid (Huang et al., 2020; Jia et al., 2019). Lastly, we have utilized 297 distribution data (139 *Ph. amurense* and 158 *Ph. chinense*) to execute the model (Figure 1; Table S1).

### 2.5 Environmental variables

In consideration of prior research and the habitat characteristics of *Phellodendron* vegetation, we selected 31 environmental factors, including bioclimate, soil, topography, and other factors, to create an ecological dataset (Table 1). The importance of environmental factors was assessed using jackknife analysis and the development of environmental variable response curves (Phillips et al., 2006), and a reiterative model‐building approach was utilized, followed by stepwise removal of minor contributing variables (Radosavljevic & Anderson, 2014; Zeng et al., 2016). Environmental datasets were used in the premodel or initial operation to minimize errors in selecting predictors and environmental factors. Ultimately, 20 of the 31 environmental factors were retained for the model.

**TABLE 1:** Environmental variables used in this study and their percentage contribution.

Hypothesis	Code	Environmental variable	Time period	*Phellodendron amurense* contribution (%)	*Phellodendron chinense* contribution (%)
Environmental energy availability	**BIO1**	**Annual mean temperature (°C)**	1970–2000	**1**	0.1
	**BIO2**	**Mean diurnal range (°C)**		0.2	**1.1**
	**BIO3**	**Isothermality (Bio3/Bio7) (°C)**		0.2	**2.1**
	**BIO4**	**Temperature seasonality**		**37.3**	0.5
	BIO5	Max temperature of warmest month (°C)		0.2	0.2
	**BIO6**	**Min temperature of coldest month (°C)**		2.6	**5.3**
	**BIO7**	**Temperature annual range (°C)**		1.3	**2.5**
	BIO8	Mean temperature of wettest quarter (°C)		0.2	0.1
	**BIO9**	**Mean temperature of driest quarter (°C)**		0.7	**3.4**
	BIO10	Mean temperature of warmest quarter (°C)		0.1	0.4
	**BIO11**	**Mean temperature of coldest quarter (°C)**		**4**	0.1
Water availability	**BIO12**	**Annual precipitation (mm)**		0.9	**5.3**
	**BIO13**	**Precipitation of wettest month (mm)**		**22.5**	2.2
	**BIO14**	**Precipitation of driest month (mm)**		1.5	**20.5**
	**BIO15**	**Precipitation seasonality (mm)**		**2.1**	0.6
	BIO16	Precipitation of wettest quarter (mm)		0.8	0.9
	BIO17	Precipitation of driest quarter (mm)		0	0
	**BIO18**	**Precipitation of warmest quarter (mm)**		**6.5**	1
	BIO19	Precipitation of coldest quarter (mm)		0.1	0.5
	**AI**	**Aridity index**	1991–2020	1.5	**36.1**
Topography factor	**ELE**	**Elevation (m)**	2019	1.1	**8.9**
	**SLO**	**Slope**		**7.4**	1.9
	**ASP**	**Aspect (°)**		0.3	**1.1**
Soil factors	SOC	Soil Organic Carbon (% wt)	2009	0.1	0
	PH	Soil pH (H_2_O)‐log (H^+^)		0.1	0.2
	ECE	Soil salinity (dS/m)		0.2	0
	REF	Soil reference bulk density (kg/dm^3^)		0.1	0.1
	**LC**	**Land cover**	2010	**1.2**	0.3
Human influence	HFI	Human footprint index	1995–2004	0.3	0.2
	**HII**	**Human influence index**		**5.1**	2.7
Productive energy	**NDVI**	**Normalized difference vegetation index**	2009–2019	0.4	**1.7**

*Note*: Energy availability and water availability factor data were all downloaded from the Worldclim 2.1 database (https://www.worldclim.org/) and the World Meteorological Organization (http://climexp.knmi.nl/); Topography factor data and Productive energy data were all from the Resource and Environment Science and Data Center (https://www.resdc.cn/); Soil factors data were downloaded from the Harmonized World Soil Database of the Food and Ariculture Organization of the United Nations (https://www.fao.org/) and the European Space Agency (https://www.esa.int/); Human disturbance data were form the Socioeconomic Data and Applications Center (https://sedac.ciesin.columbia.edu/). The parameters selected for the final model are in bold.

Similar to previous studies on vegetation dynamics (Pinedo‐Alvarez et al., 2019; Yang et al., 2019; Ye et al., 2021), we used general circulation model (GCM) predictions under Representative Concentration Pathway (RCP) scenarios to estimate the impact of climate change on species distribution. We selected the BCC‐CSM2‐MR model, which is widely used in the Middle East Asia region and has better simulation ability for East Asian climate, especially temperature, compared to other climate models (Bingrui et al., 2022; Wu, 2020). To investigate the dynamics of the target, we used current climate normals (average for 1970–2000) and future climate projections for the 2050s (average for 2041–2060) and 2070s (average for 2061–2080), utilizing representative shared socioeconomic pathways (SSPs) and concentration pathways (RCPs) for SSP2‐SSP245 (SSP245) and SSP5‐SSP585 (SSP585). The ArcGIS software was used to unify the coordinate system (WGS_1984) and resolution (2.5′ × 2.5′).

### 2.6 Optimization of model parameters and evaluation

The intricacy and overfitting of the MaxEnt model are closely linked to the regularization multiplier (RM) and FC parameter settings (Kass et al., 2021; Muscarella et al., 2014). Selecting the optimal values for these two parameters can significantly enhance the prediction accuracy of the MaxEnt model (Cobos et al., 2019; Phillips & Dudík, 2008). To ensure the MaxEnt model attains the most outstanding predictive performance, we initially employed 75% of the sample data for model training and reserved 25% for model evaluation (Li, Chang, et al., 2019; Radosavljevic & Anderson, 2014). We employed a comprehensive approach to model selection by integrating the MaxEnt algorithm with the kuenm package in the R software. We scrutinized a total of 1160 prospective models, consisting of 40 distinct configurations for the regularization multiplier, ranging from 0.1 to 4 in increments of 0.1, and 29 feature class combinations (Cobos et al., 2019). The parameter models with statistically significant and omission rates ≤5% were chosen (Li, Li, et al., 2018). Finally, the model with the lowest AICc value was screened based on the Akaike information criterion correction (AICc), AUC_DIFF_ (difference between training and testing AUC) and OR_10_ (10% training omission rate) as the optimal FC and RM parameter settings (Phillips et al., 2006; Warren & Seifert, 2011). The AICc value provides an excellent indication of MaxEnt model performance, while AUC_DIFF_ and OR_10_ measure how well the model overfits the species distribution points. The model with the most negligible AICc value (delta AICc = 0) is regarded as the best model (Osorio‐Olvera et al., 2020; Zhao et al., 2021).

In accordance with the optimal models FC and RM parameter values, we incorporated species geographic distribution (CSV format) and environmental factors (ASC format) data. We generated 10,000 random backdrop points and executed 5000 iterations with 10 repetitions (Logistic format). To gauge the accuracy of our model predictions, we utilized a threshold‐independent receiver operating characteristic (ROC) and an area under the receiver operating curve (AUC) (Aidoo et al., 2022; Guisan & Thuiller, 2005). The evaluation metric levels of the model are shown in Table 2. AUC values closer to 1 suggest more excellent model performance (Li et al., 2020; Santana et al., 2019).

**TABLE 2:** MaxEnt model prediction accuracy evaluation.

Name	Range	Description
AUC	0.50 ≤ AUC < 0.60	Failing
	0.60 ≤ AUC < 0.70	Poor
	0.70 ≤ AUC < 0.80	Good
	0.90 ≤ AUC ≤ 1.00)	Excellent

### 2.7 Changes in the spatial pattern of the potential habitat and their centroid of shift

Under several climatic scenarios, a map of species presence/absence and geographic centroid movement was created by converting continuous probability data into binary predictions using a threshold value. The results of the MaxEnt model prediction file were loaded into ArcGIS software to calculate the constant probability value of each grid species and the habitat suitability index of species, which indicates habitat suitability and distribution probability of species (0–100 percent) (Phillips et al., 2006; Phillips & Dudík, 2008). Then, an ArcGIS spatial analysis tool was utilized to determine the appropriateness of all known distribution spots on the projected distribution map. These values standard deviation and average was then calculated, and *p* = *μ*−*σ* is selected as the quasi‐threshold (Guan et al., 2019; Huang et al., 2019). Based on the maximum training sensitivity and specificity criterion, the outputs of the model prediction are checked and confirmed against the actual distribution of species (Davies et al., 2009; Jiménez‐Valverde & Lobo, 2007). The final threshold is determined. This criterion maximizes the trade‐off between specificity and sensitivity using training data, making it one of the most effective threshold selection approaches (Li, Li, et al., 2018; Wen et al., 2022). The probability values of species present in each grid of the MaxEnt prediction result file were binarized by the “Reclassify” tool according to the obtained threshold values. The predicted probability above the threshold value was “1” (named suitable habitats) and “0” (named unsuitable habitats), respectively (Jia et al., 2019; Li, Zhang, et al., 2019). Next, the “Distribution Changes Between Binary SDMs” script in the SDM toolbox was used to calculate the distribution changes between periods to determine the suitable species distribution areas. Areas that remain unchanged, areas of future expansion, and areas of future contraction are defined as retention fitness area (1 → 1), new fitness area (0 → 1), and loss fitness area (1 → 0) (Li, Chang, et al., 2019; Santana et al., 2019). Lastly, we calculated temporal changes in the geographic distribution centers of species using the “centroid change (line)” script in ArcGIS software, detecting overarching trends in suitable habitat distribution areas for species (Hu et al., 2015; Warren & Seifert, 2011).

### 2.8 Analysis of multivariate environmental similarity surface (MESS) and most dissimilar variable (MoD)

We employed the multivariate environmental similarity surface technique to compute a range of predictive variables (*V*_1_, *V*_2_, *V*_i_…) and a set of reference points to assess the extent of a climatic anomaly and the primary factors that bring about alterations in the spatial distribution of species under forthcoming climate change scenarios (Wen et al., 2022). Within the reference graph layer, mini_i_ and maxi_i_ represent the minimum and maximum values of the environmental variable *V*_*i*_. *P*_*i*_ corresponds to the value of the environmental variable *V*_*i*_ at a specific point *P* on the reference graph layer in a certain period (Elith et al., 2010). The *P* point's multivariate similarity value denotes the lowest value of each variable's similarity, signifying the variable with the highest degree of abnormality (Guo et al., 2022).

*F*_*i*_ indicates the proportion of points within the research region where the environmental variable *V*_*i*_ is less than *P*_*i*_. If *F*_*i*_ = 0, the MESS is 100 (*P* − mini)/(maxi − mini); if 0 < *F*_*i*_ ≤ 50, the MESS is 2*F*_*i*_; if 50 < *F*_*i*_ ≤ 100, the MESS is 2 (100 − *F*_*i*_); and if *F*_*i*_ = 100, the MESS is 100 (maxi − 100) / (maxi − mini) (Jia et al., 2019; Li et al., 2020). The *P* point's value is negative when at least one variable exceeds the range of the environmental variable of the reference point established during a specific period, which is known as an abnormal climatic point. The lower the value of the *P* point, the higher the degree of climate anomaly, and the greater the value, the larger the degree of climatic anomaly. When *P* equals 100, the climate is considered normal and compatible with the reference variable layer.

## 3 RESULTS

### 3.1 Evaluation of model optimization

**FIGURE 3:**
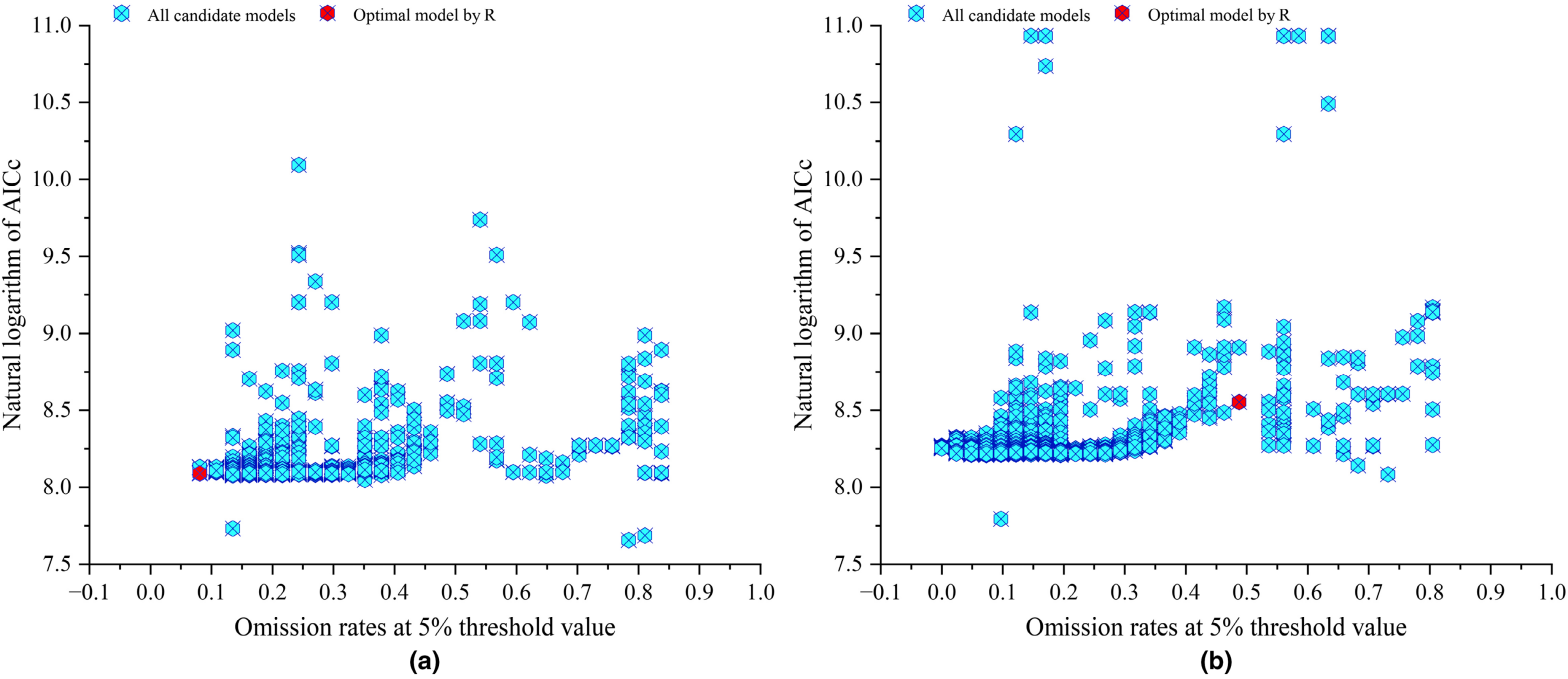
Selected optimal parametric models by R Kuenm of *Phellodendron amurense* (a) and *Ph. chinense* (b). Red dot represents the model parameter with AICc value equal to 0.

Our study has discovered that utilizing optimized model parameters can effectively reduce the likelihood of model overfitting and enhance the accuracy and confidence of the model results. Figure 3 and Table 3 demonstrate the changes in the MaxEnt models' performance when different combinations of RM and FC were used after selecting the environmental variables, indicating the filtered FC and RM parameters for the MaxEnt models with minimum delta AICc values (equal to 0). The kuenm package of R was used to select the two optimal parameters of the MaxEnt models (*Ph. amurense‐a* and *Ph. chinense‐b*). Their Mean.AUC values were 0.973 and 0.967, Mean.AUC_DIFF_ were 0.131 and 0.014, and Mean.OR_10_ values were 0.104 and 0.111, respectively (Table 3). The Mean.AUC values of the optimal parameter MaxEnt models for *Ph. amurense‐a* and *Ph. chinense‐b* improved by 13.76% and 11.09%, respectively, over the Mean.AUC values of the default parameter models (*Ph. amurense‐A* and *Ph. chinense‐B*). Similarly, Mean.OR_10_ values decreased by 49.76% and 53.36% over the default parameters. Optimizing the model parameters is necessary. Therefore, the model parameters of *Ph. amurense*‐a and *Ph. chinense*‐b were ultimately utilized to predict the distribution of suitable habitats for *Ph. amurense* and *Ph. chinense* in this study (Table 3).

**TABLE 3:** Evaluation metrics of optimal parametric models and default parametric models.

Type	Mode	FC	RM	Delta. AICc	Mean. AUC	Mean. AUC_DIFF_	Mean. OR_10_
Optimal parametric models	*Phellodendron amurense*‐a	QH	3.7	0	0.973	0.131	0.104
	*Ph. chinense*‐b	Q	1	0	0.967	0.014	0.111
Default parametric models	*Ph. amurense*‐A	LQHPT	1	325.197	0.857	0.147	0.207
	*Ph. chinense*‐B	LQHPT	1	689.028	0.834	0.021	0.238

### 3.2 Environmental variable importance

In our study, an internal jackknife test of factor importance for the two optimized models (*Ph. amurense‐a* and *Ph. chinense‐b*) revealed that temperature seasonality (BIO4, 37.3% of variation), precipitation of the wettest month (BIO13, 22.5% of variation), slope (SLO, 7.4% of variation), precipitation of the warmest quarter (BIO18, 6.5% of variation), and the Human Influence Index (HII, 5.1% of variation) were the most significant contributors to the distribution model of *Ph. amurense*, with an accumulative contribution of 78.8% (Table 1). In contrast, the major contributors to the distribution model of *Ph. chinense* were the aridity index (AI, 36.1% of variation), precipitation of the driest month (BIO14, 20.5% of variation), elevation (ELE, 8.9% of variation), minimum temperature of the coldest month (BIO6, 5.6% of variation), and annual precipitation (BIO12, 5.3% of variation), with an accumulative contribution of 76.4% (Table 1).

### 3.3 The distribution of *Phellodendron* vegetation under the current climate

**FIGURE 4:**
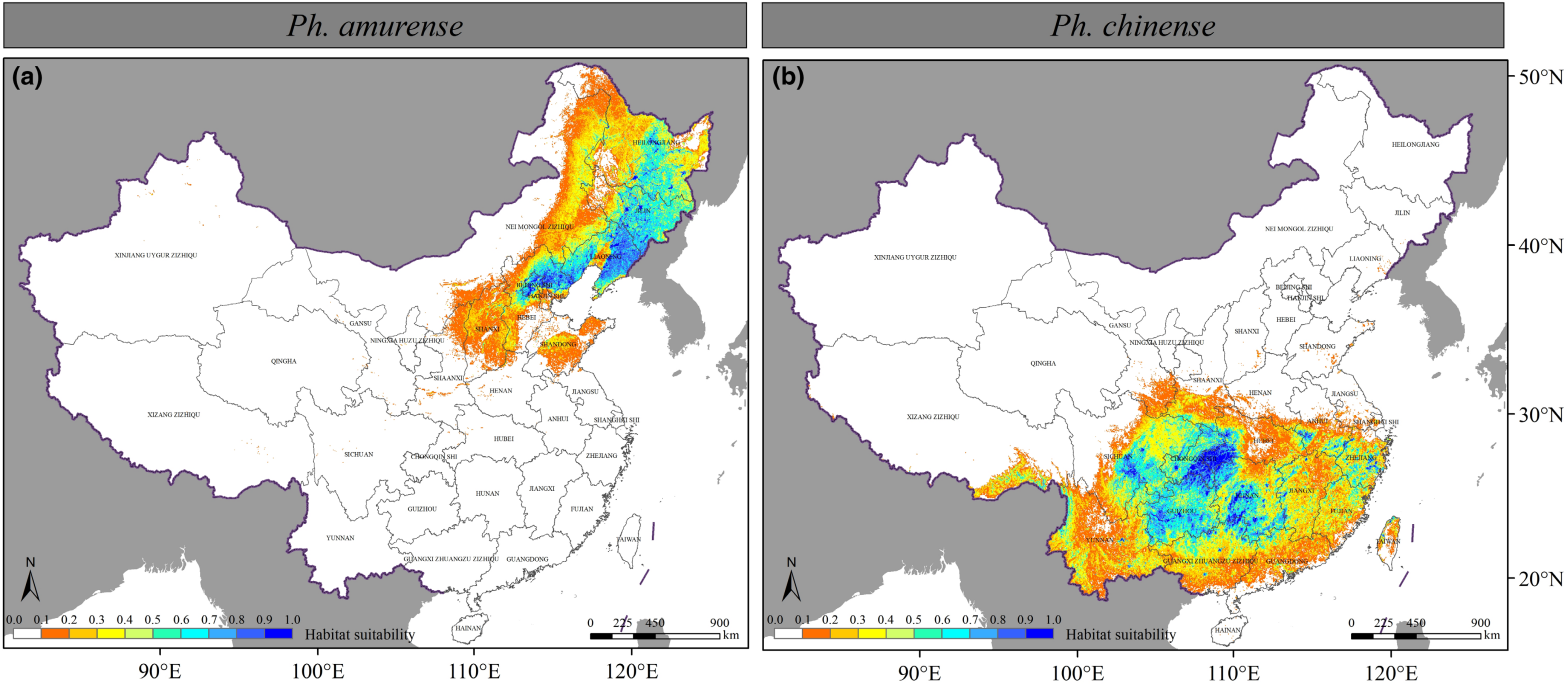
Current habitat probability maps of *Phellodendron amurense* (a) and *Ph. chinense* (b).

Using two optimized MaxEnt models driven by typical climate data (averaged from 1970 to 2000), our simulations predicted the distribution of suitable habitats for the two *Phellodendron* species in China (Figure 4). Potential habitats for *Ph. amurense* were highly concentrated in the Northeastern China Plain, North China Plain, Changbai Mountains, and Xiaoxingan Mountains (Figure 4a), while potential habitats for *Ph. chinense* were concentrated in the Sichuan Basin, Yunnan‐Kweichow Plateau, and Southeast Hills (Figure 4b). We calculate the area of the binary distribution map for each species based on the maximum training sensitivity and specificity of the presence probability maps for the two species. Our results showed that the current suitable habitat area for *Ph. amurense* is 62.89 × 104 km^2^, with 96.12% distributed in Heilongjiang, Jilin, Liaoning, Hebei, and Neimongol (Figure 4a). For *Ph. chinense*, the current suitable habitat area is 70.71 × 10^4^ km^2^, with 79.32% distributed in Chongqing, Guizhou, Hunan, Sichuan Hubei, and Guangxi (Figure 4b).

### 3.4 Future changes in suitable habitat areas of *Phellodendron* vegetation

**FIGURE 5:**
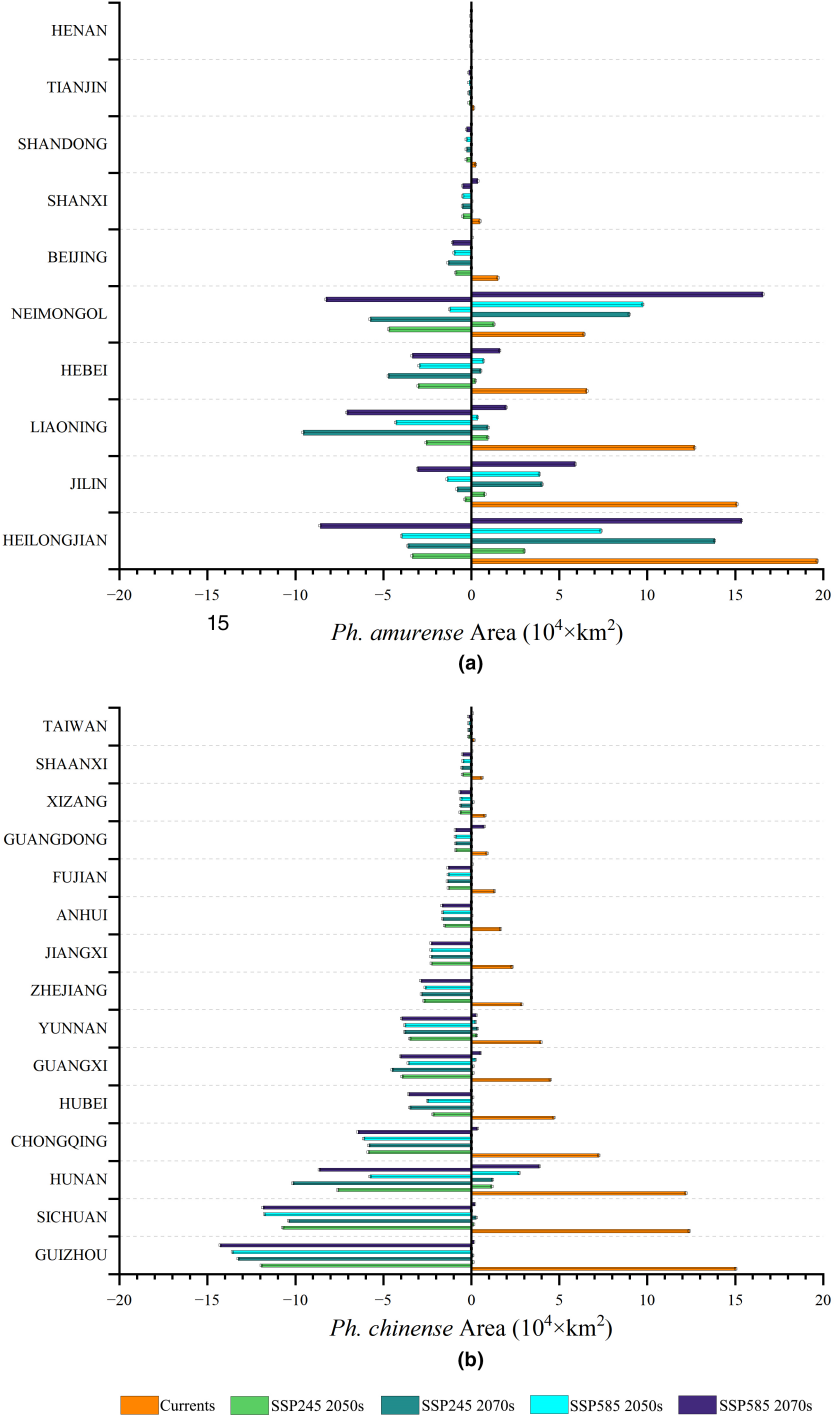
Suitable habitat change of *Phellodendron amurense* (a) and *Ph. chinense* (b) in 2050s and 2070s under different future climate scenarios (SSP245 and SSP585).

**FIGURE 6:**
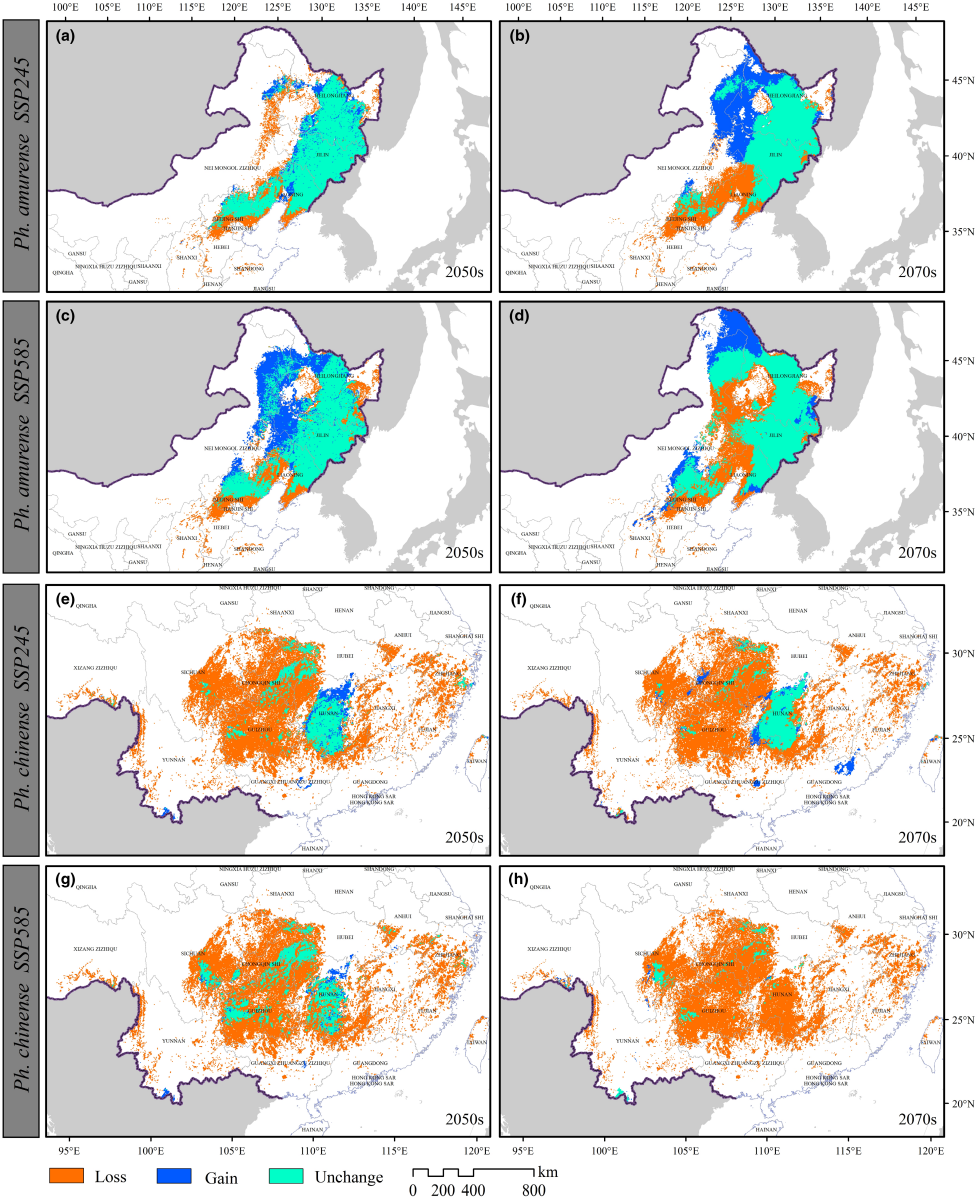
Spatial changes in the potential habitat of *Phellodendron amurense* (a–d) and *Ph. chinense* (e–h) under the SSP245 (a, b, e, f) and SSP585 (c, d, g, h) scenarios by the 2050s (a, c, e, g) and 2070s (b, d, f, h).

The dynamics of the potential habitat areas of the two species would show distinct tendencies in the 2050s and 2070s (Figure 6). We estimated that the potential suitable habitats of *Ph. amurense* will decrease to 53.37 × 10^4^ km^2^ in the 2050s and then will increase to 64.53 × 10^4^ km^2^ in the 2070s under the SSP245 scenario (Figure 5a). The expansion of *Ph. amurense* will be more prominent under the SSP585 scenario, for which the potential suitable habitat area will increase to 69.24 × 10^4^ km^2^ in the 2050s and then will increase to 72.43 × 10^4^ km^2^ in the 2070s (Figure 5a). Contrarily, the potential suitable habitat area of *Ph. chinense* will decrease by 75.13% (2050s) and 80.39% (2070s) under the SSP245 scenario (Figure 5b), and will further decrease by about 76.57% (2050s) and 84.22% (2070s) under the SSP585 scenario (Figure 5b).

By comparing the current and future suitable habitats (Figures 4 and 6), we evaluated the potential redistributions of the two *Phellodendron* species in reaction to climate change in the 2050s and 2070s under the SSP245 and SSP585 scenarios. In the two future climate scenarios, the loss and increase of suitable habitat area range for *Ph. amurense* will be 15.81 × 10^4^–32.33 × 10^4^ km^2^ and 6.28 × 10^4^–41.88 × 10^4^ km^2^, respectively, mainly in Liaoning, Inner Mongolia, Heilongjiang, and Jilin Province (Figures 5b and 6a–d). In contrast to *Ph. amurense*, *Ph. chinense* will lose a more suitable habitat (55.71 × 10^4^–63.09 × 10^4^ km^2^) under the two future climate scenarios. The disappearance of habitats suitable for *Ph. chinense* will mainly concentrate in Guizhou, Sichuan Hunan and Yunnan (Figures 5b and 6e,f).

### 3.5 Centroid of potential habitats and their spatial shift in the future

**FIGURE 7:**
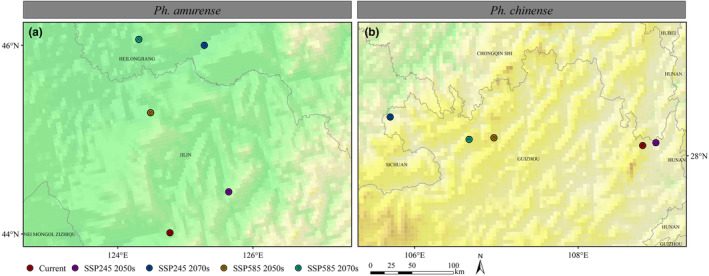
Shifts in the potential habitats of *Phellodendron amurense* (a) and *Ph. chinense* (b) under different climate change scenarios. Dots with different colors indicate the centroids of suitable habitats under current climate and different future climate scenarios.

By calculating and comparing the binary distribution maps of two *Phellodendron* species at different periods, we obtained the centroid of the potential habitat of each species (Figure 7). Currently, the centroid of *Ph. amurense*'s potential habitat is located at 124°48′ E/43°42′ N in western Jilin province (Figure 7a). Under the SSP245 scenario, the centroid will shift 100.32 km northeastward by the 2050s and then shift 146.09 km northward by the 2070s. Under the SSP585 scenario, the centroid might shift farther northward (Figure 7a). The geometric center of the current potentially suitable habitat of *Ph. chinense* is located northeastward of Guizhou Province (108°50′ E/28°7′ N) (Figure 7b). By the 2050s, the centroid of *Ph. chinense*'s potential habitat will shift 16.24 km northeastward and then turn 319.88 km westward by the 2070s under the SSP245 scenario (Figure 7b). Under the SSP585 scenario, the centroid is expected to shift 176.84 km westward and then turn 28.68 km southwestern (Figure 7b).

### 3.6 Multivariate environment similarity surface (MESS) and most dissimilar (MoD) variable under different climatic scenarios

**FIGURE 8:**
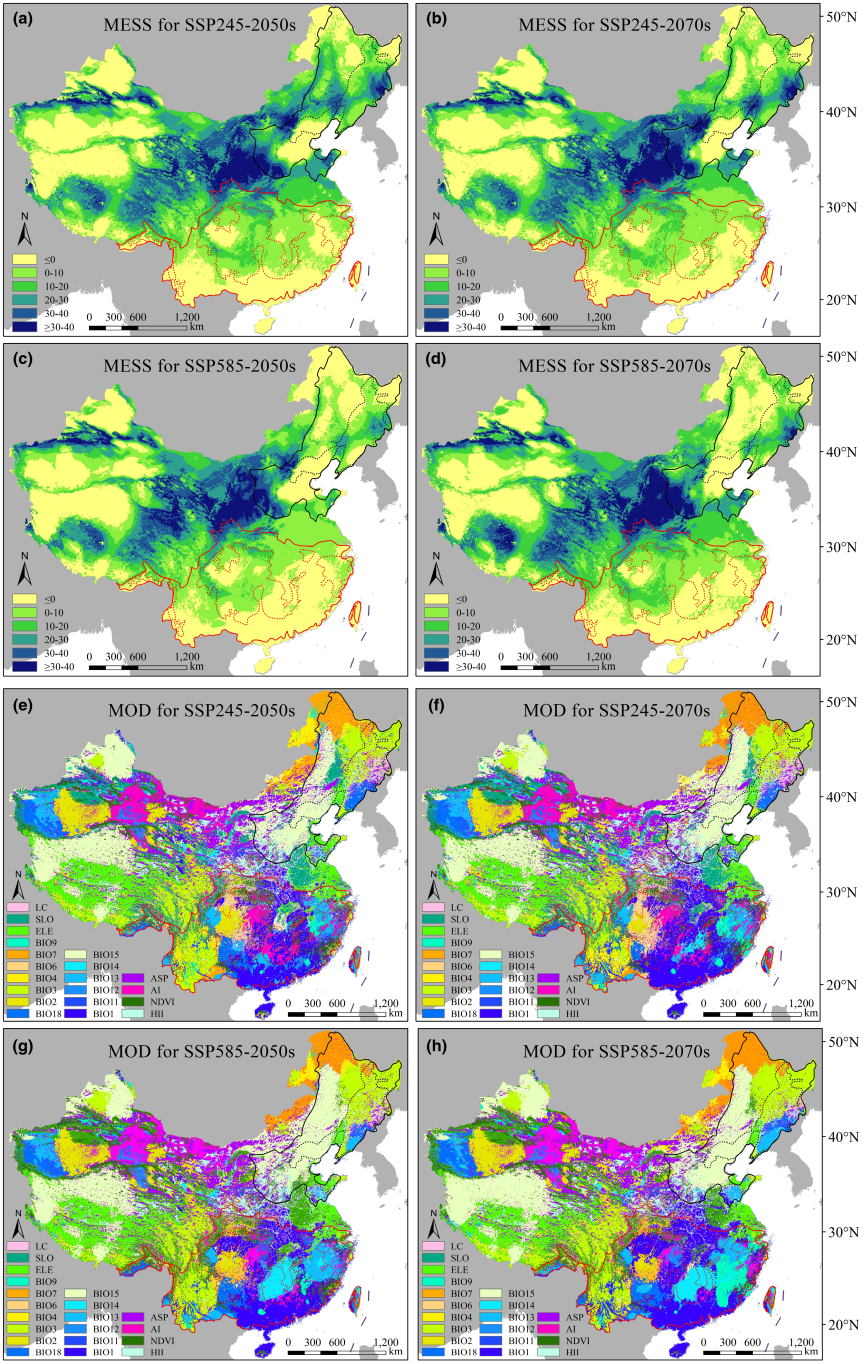
Multivariate environmental similarity surface (MESS, a–d) and most dissimilar (MoD, e–h) variable analysis of *Phellodendron amurense* and *Ph. chinense* under the SSP245 (a, b, e, f) and SSP585 (c, d, g, h) scenarios by 2050s (a, c, e, g) and 2070s (b, d, f, h). The solid line represents the species' maximum suitable habitat, while the dashed line represents the species' core suitable habitat.

Based on the current environmental factor layers, we explored the degree of climatic anomaly and the changes of critical environmental factors under future climate change scenarios (Figure 8). The average multivariate similarities to the 139 current distribution points of *Ph. amurense* were 5.53 to 9.01 under the two future climate change scenarios (Figure 8a–d). The multiple similarity value was the lowest, and the degree of the climatic anomaly was most outstanding in the SSP585‐2070s climate scenario. In contrast, the SSP245‐2050s climate scenario had the highest multiple similarities and the lowest degree of climate anomaly (Figure 8a–d). The most different variables in the current potential habitat for *Ph. amurense* were BIO3, BIO7, and BIO15 (Figure 8e,f). Unlike *Ph. amurense*, the 158 current distribution points of *Ph. chinense* showed multivariate similarities ranging from 7.33 to 14.75. The SSP585‐2050s climatic scenario had the highest multiple similarities and the lowest degree of climate anomaly, whereas the SSP245‐2050s had the lowest similarity value and the highest degree of climate anomaly (Figure 8a–d). The most different variables in the current potential habitat for *Ph. chinense* were BIO1, BIO2, and BIO14 (Figure 8e,f).

## 4 DISCUSSION

### 4.1 The limitation of the model and the necessity of optimizing model

Known as the MaxEnt model is a powerful tool for predicting potential species distributions, which can inform applied ecology (Araujo et al., 2005; Gaston, 2000). However, it suffers from limitations, such as low transferability and discrepancies between simulation and actual species distribution, leading to inaccurate inferences (Jia et al., 2019; Radosavljevic & Anderson, 2014). Reliable and precise simulation results depend on three critical factors: (1) MaxEnt model parameter selection (Huang et al., 2020); (2) uniformity of species sample collection range and coverage (Xie et al., 2021); (3) precision of environmental factors sources (Ye et al., 2021). Notably, the default MaxEnt model usually retains a random subset of data for modeling and then uses the AUC value to evaluate the prediction ability and accuracy of the model, which has certain defects (Qiao et al., 2015). This leads to overfitting and a decrease in transferability when using the default parameters to predict possible species distribution suitability (Warren & Seifert, 2011).

In this study, we selected the optimal model parameters using the kuenm package of R software and referring to AICc, AUC_DIFF,_ and OR_10_. (Figure 3; Table 2). By comparing the AUC, AICc, AUC_DIFF,_ and OR_10_ of the optimal model and the default model, we found that using optimal model parameters can effectively improve the accuracy of the model prediction. Notably, our optimal model's predicted distribution had a consensus with the observed *Phellodendron* vegetation distribution in the field (Yang et al., 2019 and Figure 1). The optimal model could predict the redistribution of two *Phellodendron* species under future temperature scenarios. Our research results confirmed that processing species sample data and optimizing parameters were necessary when using the MaxEnt model to study species habitat distribution.

### 4.2 Environmental explanations for the potential distribution of *Phellodendron* vegetation

Previous research has suggested that climate change is a dominant factor in species extinction rates, habitat distribution, and vegetation type changes (Mittelbach et al., 2007). Similar to previous findings (Huang et al., 2018), our study showed that temperature and precipitation are major factors controlling *Phellodendron* population distribution (Table 1). Among environmental factors, BIO4 and BIO13 contributed the most (37.3% and 22.5%, respectively) to the MaxEnt model for *Ph. amurense*, followed by SLO (7.4%). For *Ph. chinense*, AI and BIO14 contributed the most (36.1% and 20.5%, respectively) to the MaxEnt model of the species, followed by ELE (8.9%). Similarly, the effects of climate factors on *Phellodendron* vegetation growth study (Yang et al., 2019) indicated that temperature was the main factor, followed by moisture for their growth. Three potential mechanisms explained the significance of temperature, precipitation, and topography. (i) As with many other plants, seasonal temperature determines the regular phenology of *Phellodendron* vegetation, while precipitation affects their water supply during the growing season (Wan et al., 2014). (ii) The intensity and duration of drought can affect photosynthesis, budding ability, and seedling survival of *Phellodendron* vegetation (Wang, Yu, et al., 2021). (iii) Temperature and water change with topographic gradient and then affect the distribution of *Phellodendron* vegetation (Fan et al., 2021; Qin et al., 2006).

### 4.3 The critical influence of climate change on the distribution of *Phellodendron* vegetation

With a projected temperature in China increase (0.6–6.3°C) by the end of the 21st century, there is concern that the forest ecosystem will move northward as a whole, and plants will migrate to higher altitudes (Ni, 2010; Pinedo‐Alvarez et al., 2019). Dramatic warming in winter and spring, the season when the seeds of *Phellodendron* vegetation germinated at low temperatures, had occurred in China in recent years (Zhang et al., 2016; Zhang, Li, et al., 2017). Climate change has been beneficial to regeneration and alleviating resource depletion of *Ph. amurense* (Duan et al., 2019).

The possible habitat of *Ph. amurense* should migrate significantly northward in China and become seriously endangered in the Liaoning and Hebei Province (Figures 6a–d and 7a). For *Ph. chinense*, it will migrate to higher elevations in the west, and lower elevation habitats will be lost (Figure 6e,f). The different reactions of these species might be attributed to their physiological differences. For example, *Ph. amurense* could retain a substantially larger photosynthetic capacity under cold stress and had a higher cold tolerance than that of *Ph. chinense* (Duan et al., 2019; Wang, Huang, et al., 2021). Global warming is more favorable for the growth and regeneration of *Ph. amurense* (Huang et al., 2018). The potential habitat area of *Ph. amurense* was predicted to increase from 2.61% (SSP245) to 153.18% (SSP585), but that of *Ph. chinense* was predicted to decrease from 80.35% (SSP245) to 84.41% (SSP585) in a warmer twenty‐first century (Figures 5 and 6). The distinct vegetation dynamic trends exhibited by these two *Phellodendron* species underscore the importance of considering their divergent climate responses. This aspect has been largely neglected in previous studies, such as those conducted by Huang et al. (2018) and Yang et al. (2019), when modeling the vegetation dynamics of these two *Phellodendron* species in China. In addition, the two hypothesis we propose was supported by by the changes in habitat area and centroid distribution of the two species.

### 4.4 Recommendations for cultivation and re‐introduction of *Phellodendron* vegetation

Widrlechner et al. (2012) use the average annual minimum temperature index to divide the chill resistance of China into 11 levels, which generating the hardiness zone map in China and successfully guiding the regional cultivation of plants. Based on the model results and the hardiness zones map, we can calculate the distribution range of *Ph. amurense* under the current climate conditions of 1–6 (−40.0 to −17.8°C), and the distribution range of *Ph. chinense* is 9–10 (−6.6 to 4.4°C). We inferred that *Ph. amurense* could be cultivated in the southeast of NeiMongol and Shaanxi; *Ph. chinense* can be planted in Zhejiang, Jiangxi, Guangdong and Fujian.

**FIGURE 9:**
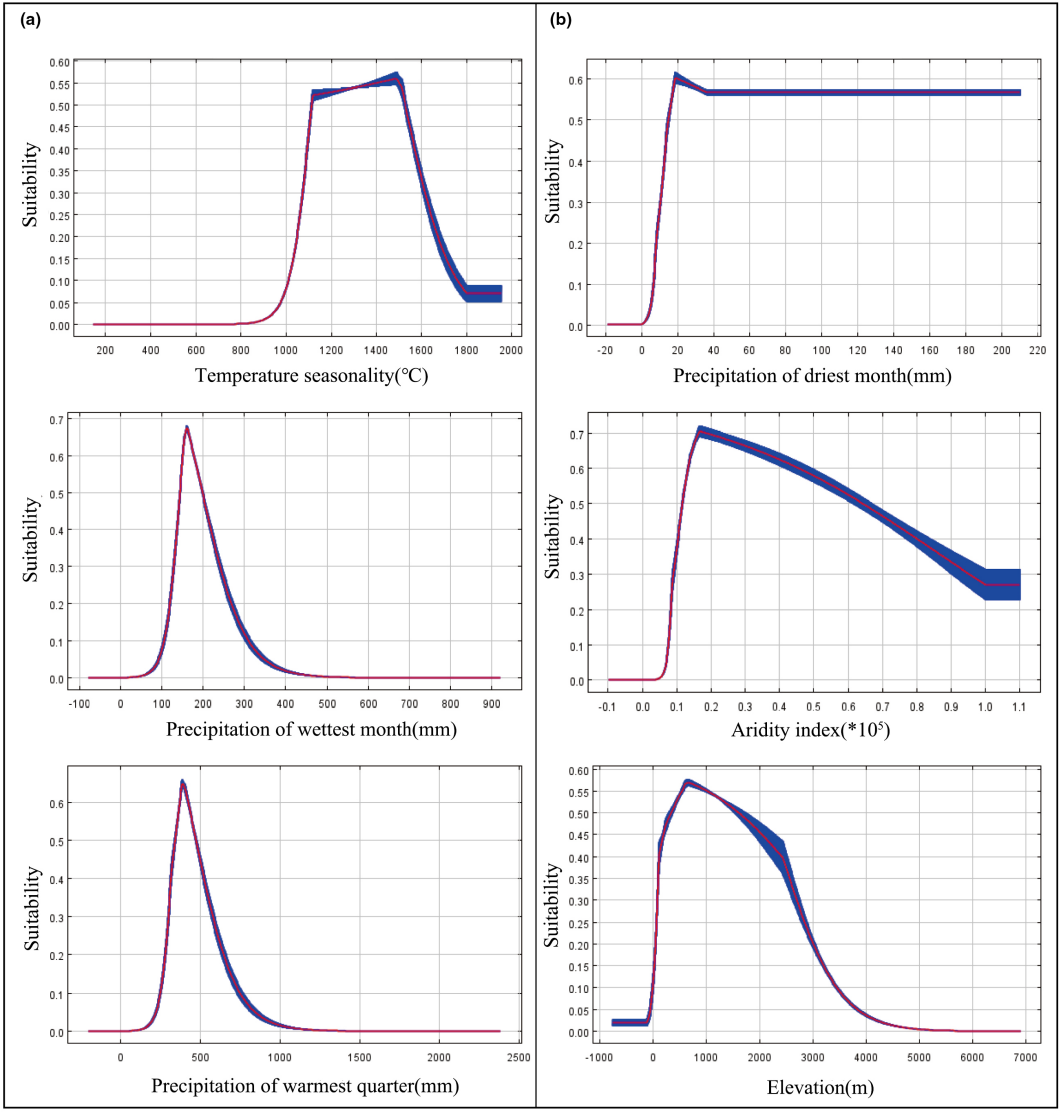
Response curves of habitat probabilities for *Phellodendron amurense* (a) and *Ph. chinense* (b) with respect to mainly three environmental factors. The response curves are presented as themeans of 10 replicates with standard deviations shown in blue.

Previous studies had concluded that the pollination of *Phellodendron* populations was easily blocked and their seeds had dormancy and postembryonic ripening, which led to poor natural regeneration (Fan et al., 2021; Zhang et al., 2016). Their seedlings were easily damaged by the stressed environmental conditions and human activities (Zhang & Chang, 2020). Therefore, they have limited geographical distribution, which was one of the crucial reasons that contributed to their endangered status. Jackknife tests resulted that hydrothermal conditions (cumulative contribution > 80%) were the main factors limiting the growth of the two *Phellodendron* species (Table 1). The reason was that the seed germination, the efficiency of *Phellodendron* photosynthesis, and soil water content were all regulated by temperature and precipitation (Li, 2013; Li, Fang, et al., 2018). Appropriate hydrothermal conditions benefited the accumulation of organic matter and seed germination of *Phellodendron*. According to the species response curve output by the model (Figure 9), *Ph. amurense* was suitable for growing in the conditions of BIO4 10.6–16.3°C, BIO13 110–280 mm, precipitation of BIO18 260–600 mm (Figure 9a). AI between 0.1 and 1, BIO14 > 15 mm, and ELE < 3000 m were suitable for *Ph. chinense* survival (Figure 9b). The multivariate environmental similarity surface and most dissimilar variable analyses revealed that climatic anomaly zones would migrate to high and low latitudes. The temperature under future global warming was the most divergent variable relating to these trends (Figure 8). The increase in the BIO3 and BIO7 improved its adaptability to high‐latitude regions. On the contrary, the increase in the BIO1 and BIO2 reduced its suitability to low‐latitude areas. Based on the above results, we considered that changes in two *Phellodendron* species habitats were mainly regulated by temperature and precipitation. It is highly recommended to strengthen dynamic monitoring of their current distribution ranges and update their endangered status in time.

### 4.5 Strengths and limitations of the study

Our research framework (Figure 2) for the potential distribution of species under climate change can be applied to many other similar plants where they urgently need to be explored the impact of climate change in suitable distribution areas. Two *Phellodendron* species records were mainly obtained from collection sites of wild *Phellodendron* vegetation specimens and field surveys, improving the authenticity and availability of data. We calibrated the environmental data layers collected from multiple national authoritative databases to ensure that they were consistent with the climate of the study area and bio‐geographical conditions of two *Phellodendron* species so that the model prediction results can play a valuable guiding role. Our study results indicated that our optimized model estimations matched the current observations of the *Phellodendron* distribution pattern and might reflect the physiological differences between the two *Phellodendron* species. These findings demonstrated that the potential habitats of the *Phellodendron* within the context of climate change were accurately predicted using our research framework.

Although we had 297 specimen records (139 *Ph. amurense* and 158 *Ph. chinense*) ‐ the most ever assembled ‐ for the two *Phellodendron* species. However, our study solely focused on the distribution of two target plant species and their climatic preferences when predicting suitable habitats. It is worth noting that the dispersal capacity of species and biological competition, such as plant seed dispersal ability (Li, Zhang, et al., 2019; Yang et al., 2021), could also influence species migration and diffusion. By considering geographic barriers and species dispersal abilities, the actual suitable range for the two target species might be narrower than the predicted habitat. Moreover, in addition, our study did not consider regions and species occurrence sites outside of China, which somewhat affects the accuracy of the model predictions. Future studies should cover the current distribution areas of both species as much as possible to improve the model's prediction accuracy and provide more accurate conservation and cultivation guidance. The type variables of environmental energy availability and water availability were a long‐term mean data regime and could not consider inter‐annual climate fluctuations (Table 1). To improve the accuracy of forecasting the natural habitat dynamics of *Phellodendron* vegetation, it may be necessary to incorporate high‐precision and high‐quality environmental data. These aforementioned aspects warrant consideration in future investigations to bolster the precision of research findings.

## 5 CONCLUSIONS

Based on 297 occurrence points of species(139 *Ph. amurense* and 158 *Ph. chinense*), 20 of 31 environment variables, and the MaxEnt model, we have developed a methodological framework to uncover the potential change in the geographical distribution of two *Phellodendron* species under climate change. Our results indicate that the optimal model, utilizing AICc, AUC_DIFF_, and OR_10_ to select significant parameters, considerably reduces overfitting and complexity, while improving prediction accuracy compared to the default model. Under the present climate conditions, temperature and precipitation are the primary factors affecting the distribution range of the two *Phellodendron* species. In contrast, hydrothermal change is the primary driver of suitable habitat redistribution of the two species in the 21st century. Additionally, due to climate change, *Ph. Amurense* potential habitat is predicted to shift northward, decreasing by 12.52% (SSP245) and increasing by 25.28% (SSP585) from its current area of 62.89 × 104 km^2^. Conversely, *Ph. Chinense* potential habitat will shift westward, resulting in a decrease of 80.39% (SSP245) to 84.22% (SSP585) from its current area of 70.71 × 104 km^2^. Our research contributes to a more comprehensive understanding of the dynamic distribution patterns of *Phellodendron* vegetation in China, driven by climate change. These findings provide valuable insights for formulating long‐term protection, regional management, re‐introduction, and sustainable use strategies for *Phellodendron* vegetation.

## AUTHOR CONTRIBUTIONS

**YangHui Zhao:** Conceptualization (lead); formal analysis (lead); investigation (lead); methodology (lead); software (lead); validation (lead); visualization (lead); writing – original draft (lead); writing – review and editing (lead). **Yafeng Wen:** Investigation (equal); validation (equal); writing – review and editing (lead). **Wenqian Zhang:** Investigation (supporting); methodology (equal); supervision (equal). **Chuncheng Wang:** Investigation (supporting); supervision (equal). **Yadan Yan:** Investigation (supporting); supervision (equal). **Siwen Hao:** Investigation (supporting); supervision (equal). **Donglin Zhang:** Conceptualization (equal); funding acquisition (lead); investigation (supporting); methodology (supporting); project administration (lead); supervision (lead); validation (lead); writing – review and editing (equal).

## FUNDING INFORMATION

This study was supported by the Key Discipline of China Forestry Bureau (Grant No. 2016 21), Double First‐class Initiative Cultivation Discipline in Hunan Province (Grant No. 2018469), Hunan Provincial Innovation Foundation for Postgraduate (Grant No. CX20220700).

## CONFLICT OF INTEREST STATEMENT

All authors declare no conflict of interest.

## Supporting information


Table S1

## Data Availability

The dataset used in this study is publicly available in Dyrad Digital Repository (https://doi.org/10.5061/dryad.wpzgmsbrq).
